# Supplementary figures and images for: Optimal occlusion uniformly partitions red blood cells fluxes within a microvascular network
Source: PLoS Comput Biol. 2017 Dec 15;13(12):e1005892. doi: 10.1371/journal.pcbi.1005892 (PMC5747476; doi:10.1371/journal.pcbi.1005892)

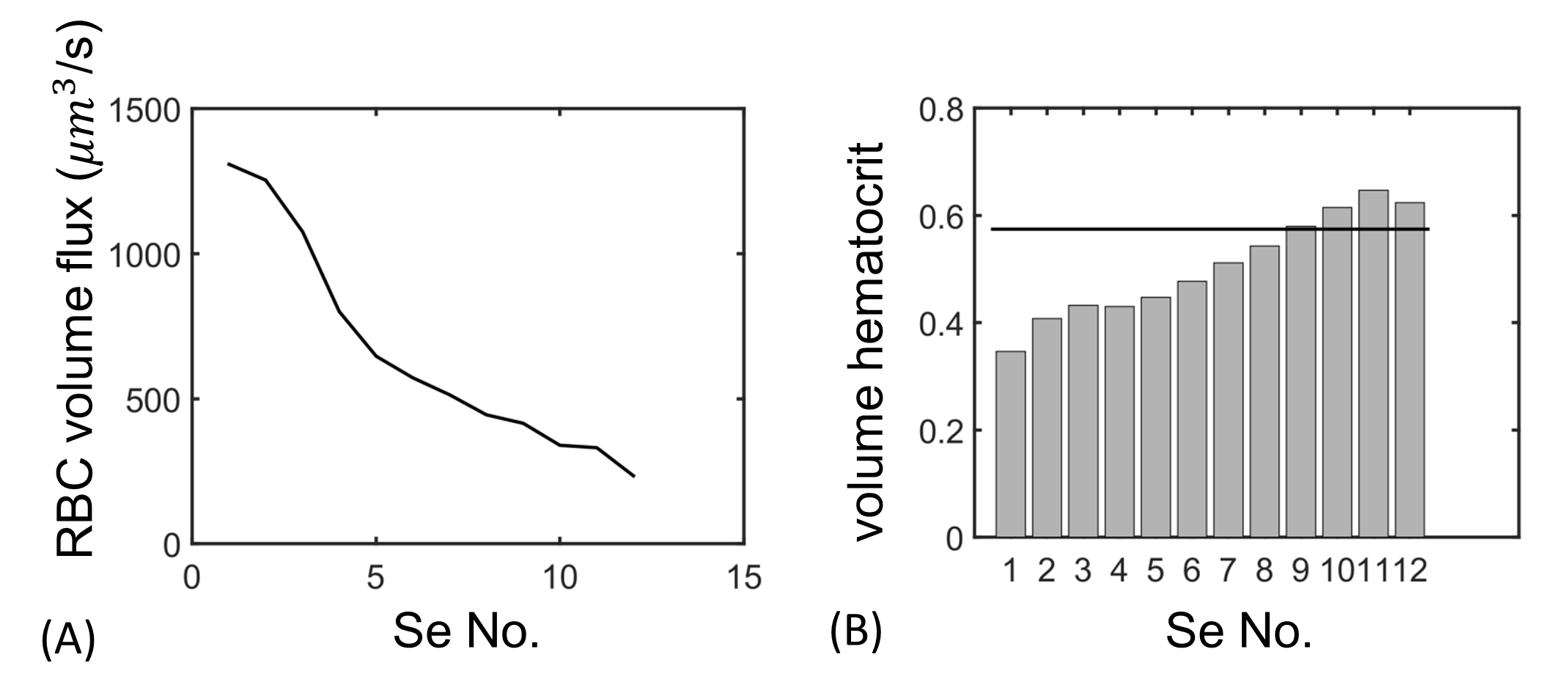

Supplement: S1 Fig — (TIF) [file pcbi.1005892.s001.tif]

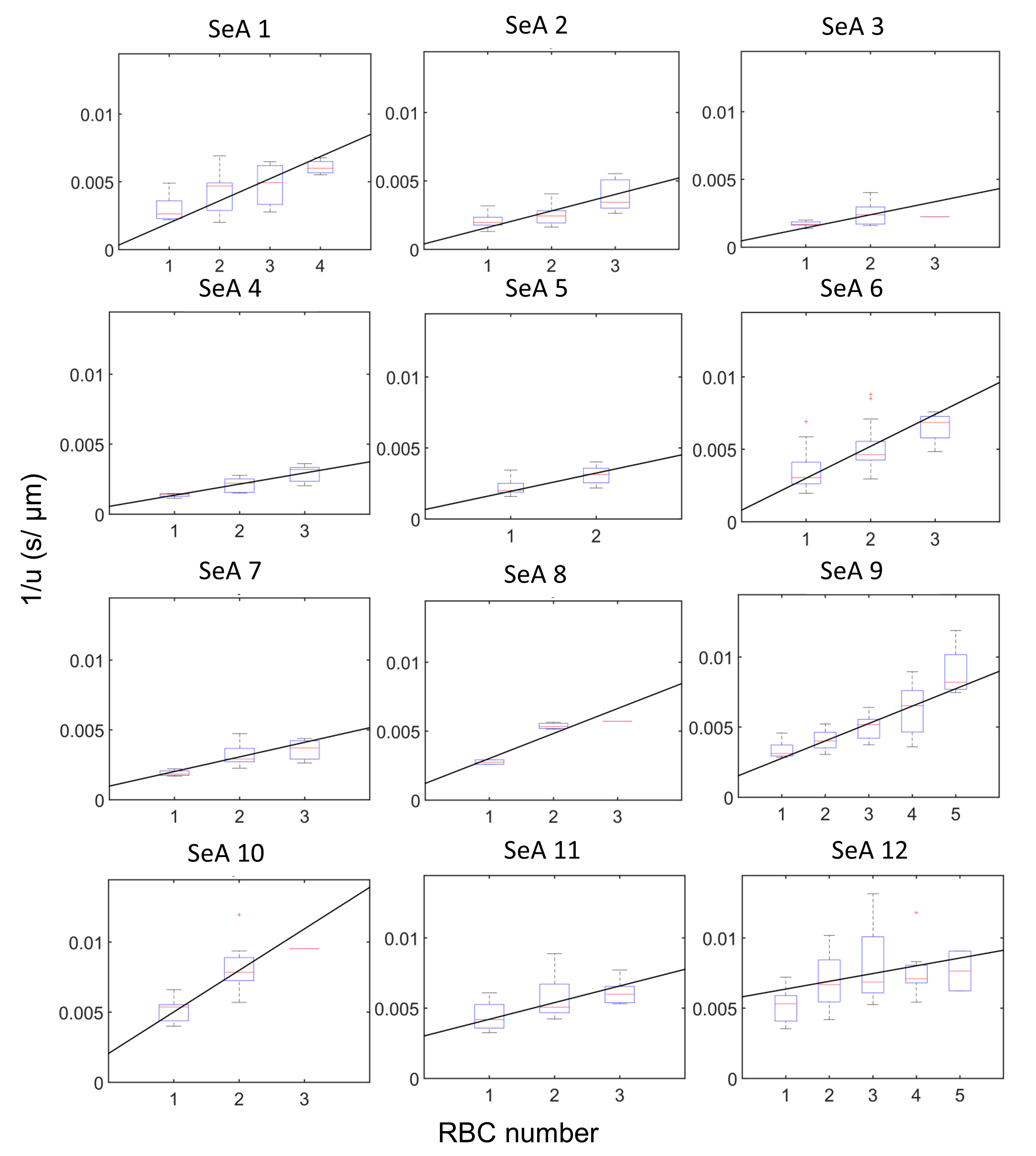

Supplement: S2 Fig — (TIF) [file pcbi.1005892.s002.tif]

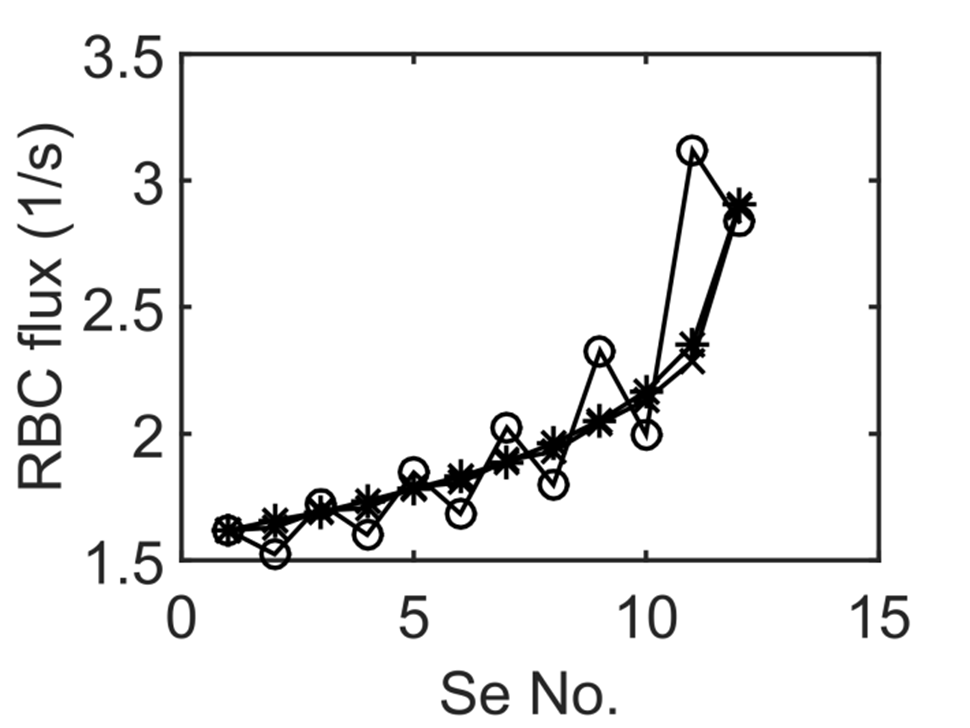

Supplement: S3 Fig — (TIF) [file pcbi.1005892.s003.tif]

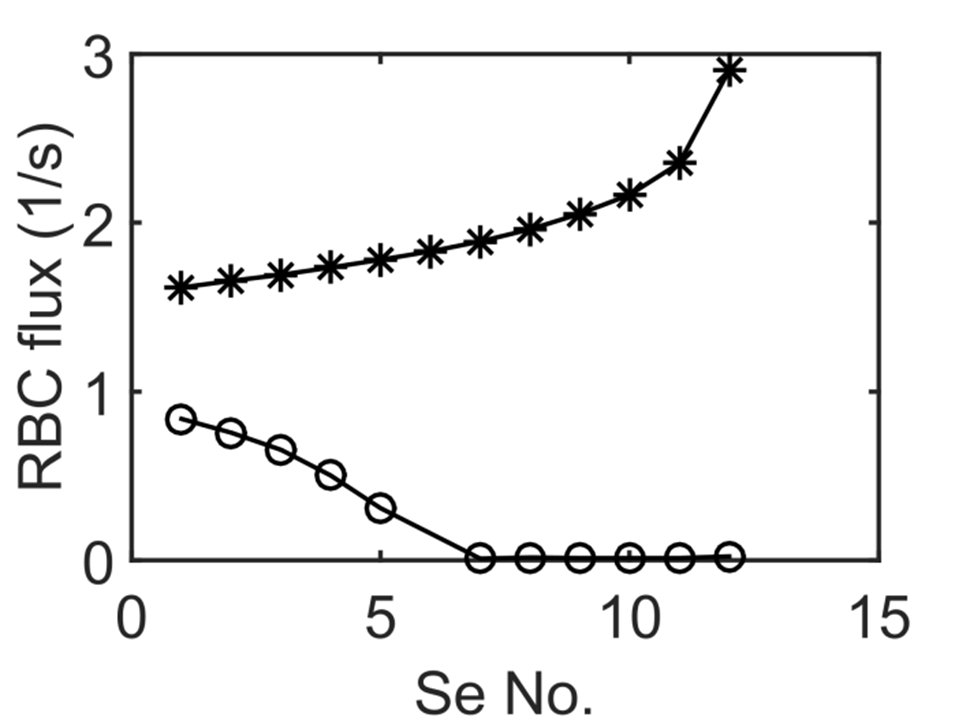

Supplement: S4 Fig — (TIF) [file pcbi.1005892.s004.tif]
