# Supplementary material for: Optimal occlusion uniformly partitions red blood cells fluxes within a microvascular network
Source: PLoS Comput Biol. 2017 Dec 15;13(12):e1005892. doi: 10.1371/journal.pcbi.1005892 (PMC5747476; doi:10.1371/journal.pcbi.1005892)
Supplement: S1 Table — (PDF) [file pcbi.1005892.s005.pdf]

i	l_i (μm)	r_i (μm)	i	l_i (μm)
1	150	5.9	2	151
3	183	7.6	4	141
5	178	6.1	6	156
7	174	6.6	8	160
9	155	6.0	10	172
11	175	6.4	12	166
13	169	5.9	14	163
15	166	6.1	16	156
17	174	5.4	18	146
19	168	6.0	20	138
21	168	4.8	22	123
23	169	3.5	24	113

TABLE S1: The lengths of all 24 vessels and radii of all 12 aorta segments in a 4dpf zebrafish embryo. The radius of capillaries is set to be the mean value 2.9 μm in Fig. 1C and Fig. S1. Vessels are numbered as in Fig. 1B (i.e. odd numbered vessels correspond to sections of dorsal aorta, even numbered vessels to intersegmental arteries).
