# Supplementary material for: Optimal occlusion uniformly partitions red blood cells fluxes within a microvascular network
Source: PLoS Comput Biol. 2017 Dec 15;13(12):e1005892. doi: 10.1371/journal.pcbi.1005892 (PMC5747476; doi:10.1371/journal.pcbi.1005892)
Supplement: S1 Text — (PDF) [file pcbi.1005892.s006.pdf]

Text S1 with Supplementary Figures S1-S4 and Table S1

Shyr-Shea Chang,[1] Shenyinying Tu,[1] Kyung In Baek,[2] Andrew Pietersen,[2] Yu-Hsiu Liu,[3]
Van M. Savage,[4,5,6] Sheng-Ping L. Hwang,[7] Tzung K. Hsiai,[2,8] and Marcus Roper[1,4]

[1]*Dept. of Mathematics, University of California Los Angeles, Los Angeles, CA 90095, USA*

[2]*Dept. of Bioengineering, School of Engineering & Applied Science, University of California Los Angeles, Los Angeles, CA 90095, USA*

[3]*Department of Life Science, National Taiwan University, Taipei, Taiwan 10617, R.O.C.*

[4]*Dept. of Biomathematics, University of California Los Angeles, Los Angeles, CA 90095, USA*

[5]*Department of Ecology and Evolutionary Biology, University of California Los Angeles, Los Angeles, CA 90095, USA*

[6]*Santa Fe Institute, Santa Fe, NM 87501*

[7]*Institute of Cellular and Organismic Biology, Academia Sinica, Nankang, Taipei, Taiwan 11529, R.O.C.*

[8]*Division of Cardiology, Department of Medicine, University of California Los Angeles, Los Angeles, CA 90095, USA*

(Dated: December 1, 2017)

Abstract

This Appendix contains the following supplementary discussions, data table and figures:

S1 Measured vessel lengths and radii within the zebrafish trunk (Table S1).

S2 Modeling oxygenation within the trunk.

S3 Incorporating phase skimming in the network model. (Figure S1)

S4 Discussion of the mean field model for a reduced network.

S5 Estimation of occlusive effects in a 4 dpf zebrafish (Figure S2)

S6 Effect of network perturbation upon red blood cell partitioning (Figures S3 and S4)

[] *To whom correspondence should be addressed: shyrsheachang@ucla.edu

S1. LENGTHS AND RADII OF TRUNK VESSELS

i	l_i (μm)	r_i (μm)	i	l_i (μm)
1	150	5.9	2	151
3	183	7.6	4	141
5	178	6.1	6	156
7	174	6.6	8	160
9	155	6.0	10	172
11	175	6.4	12	166
13	169	5.9	14	163
15	166	6.1	16	156
17	174	5.4	18	146
19	168	6.0	20	138
21	168	4.8	22	123
23	169	3.5	24	113

TABLE S1: The lengths of all 24 vessels and radii of all 12 aorta segments in a 4dpf zebrafish embryo. The radius of capillaries is set to be the mean value 2.9 μm in Fig. 1C and Fig. S1. Vessels are numbered as in Fig. 1B (i.e. odd numbered vessels correspond to sections of dorsal aorta, even numbered vessels to intersegmental arteries).

S2. MODELING OXYGEN PERFUSION

In the early stages of embryogenesis, diffusion of oxygen through the zebrafish's skin is generally sufficient to supply zebrafish tissues with oxygen[1]. However, circulatory system looping defects are typically lethal by 17.5 d.p.f.[2], suggesting oxygen transport by the circulatory system contributes to oxygen supply relatively early in embryonic development. To determine whether diffusion of oxygen through tissues might compensate for unequal partitioning of oxygen supply between micro-vessels, we directly model the diffusive transport of oxygen through the zebrafish torso using a reaction-diffusion model[3]. Within the zebrafish trunk the oxygen partial pressure, P, obeys a reaction diffusion equation:

$$D\alpha\nabla^2 P = -C + S \tag{S1}$$

where S represents the distribution of oxygen supply from the blood, and C the rate of oxygen consumption per volume of tissue, α is the solubility of oxygen and D is the diffusivity of dissolved oxygen. We solved this Partial Differential Equation by creating a Finite Element Model with first order tetrahedral elements implemented in Comsol Multiphysics (Comsol, Los Angeles). We extracted the geometry of the trunk muscles from the Zebrafish Anatomy Portal [4], and the distribution of intersegmental vessels within the trunk from the Zebrafish Vascular Atlas [5]. The parameter $D\alpha$, sometimes called the oxygen permeability, was measured by [3] to be: $D\alpha = 8 \times 10^{-14}$ m^2/(s mmHg). We modified the oxygen consumption rate found by [3] to: $C = 5.1 \times 10^{-4} P/40$ ml oxygen/(ml tissue mmHg). This formula agrees with the rate measured by [3] when $P = 40$ mmHg, but is smaller at lower oxygen partial pressures, representing the regulation of tissue oxygen consumption with oxygen availability. The source term represents the rate of oxygen release from blood, and is compactly supported on the intersegmental vessels. We used the following conversion factors: assuming that when a red blood cell enters a vessel with all of its hemoglobin molecules bound to oxygen, and that all of this oxygen is released, the total oxygen release from each intersegmental vessel can be computed from:

$$\text{rate of oxygen release (ml/s)} = 1.39 \times 10^{-10} \times \text{flow rate (in cells/s)} \tag{S2}$$

we distribute this flux uniformly across the length of each segmental artery. We apply no-flux boundary conditions on the boundaries of the trunk. By applying this boundary

condition, our model represents only the contribution of oxygen transport in the circulatory system to tissue oxygen levels. Oxygen diffusing through the skin of the fish will increase the oxygen partial pressure everywhere by a constant amount and will ensure that all tissues are sufficiently oxygenated, but does not affect the absolute differences in partial pressure that our model is designed to measure. The results of this calculation are shown in Figure 1D in the main text.

S3. INCORPORATING PHASE SKIMMING IN THE NETWORK MODEL

In our models for red blood cell transport within the trunk vasculature, we assume that red blood cells divide at branching points in the same ratio as whole blood. In fact, red blood cells are more likely to enter the larger of the two daughter vessels at a branching point than would be expected based on the ratio of fluxes [6] an effect known as phase skimming. We use the mathematical model developed in [6], to see whether phase skimming can lead to uniform distribution of red blood cells between SeAs.

Incorporating the phase skimming model of [6] significantly alters hematocrits between different SeAs. However the overall change in fluxes is much smaller than the predicted 11-fold decrease in flux between the first and last SeA. The ratio of SeA diameters to DA diameters ranges from 0.39 for the first SeA to 0.85 for the last SeA (Table S1) and velocities are similar over the entire length of the DA. Due to the increasing in the ratio of SeA to DA radius with distance from the heart the red blood cells are more likely to enter SeAs further from the heart than those closer. This leads to a 2-fold increase of SeA hematocrit (Fig. S1B) from $Hct = 0.35$ in the first SeA to $Hct = 0.65$ in the twelfth SeA. However the increase in Hct is not enough to compensate for the 11-fold change in whole blood fluxes between first and last SeA: when phase skimming was incorporated into the model we still saw a 5 fold decrease in red blood cell fluxes between first and last SeAs (Fig. S1A).

The phase skimming model of [6] parameterizes observations blood flows in the rat mesentery. The difference between the sizes, shapes and mechanical properties of zebrafish red blood cells and rat red blood cells may mean the the model is not quantitatively accurate for the zebrafish microvasculature. It is therefore worth asking whether any model for phase skimming could account for the experimental observations. Recall that the model predicts an 11-fold difference between the red blood cell fluxes in the first and last of the SeAs. To compensate for this difference the hematocrit in the last SeA would need to be 11 times larger than the hematocrit in the first SeA. Such a large difference in hematocrit would be easy to detect, and is not supported by our observations, nor are such large differences observed in any of the mammalian vessels measured by [6].

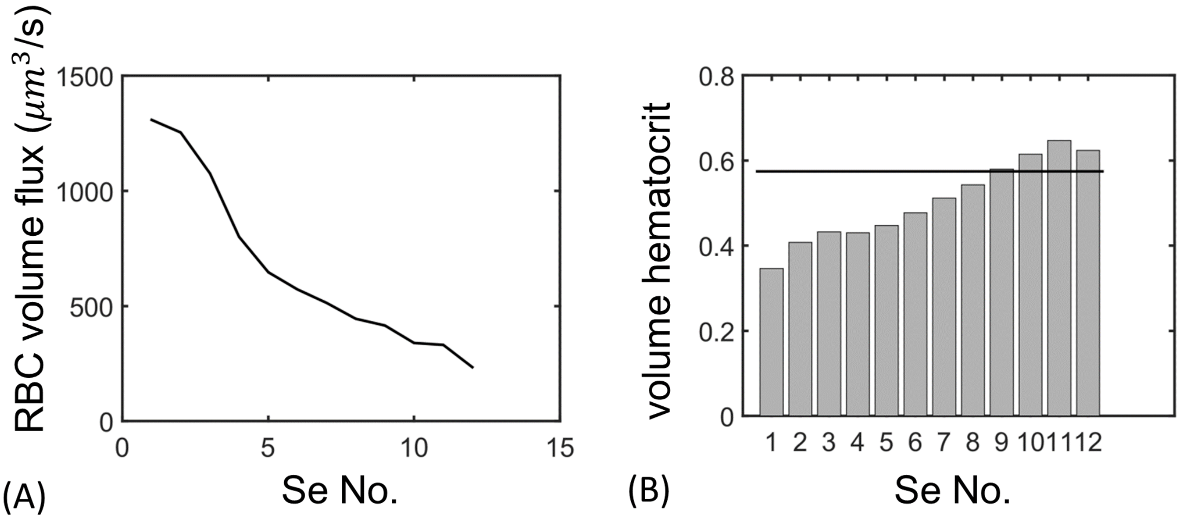

FIG. S1: Incorporating phase skimming into the model does not produce uniform red blood cell fluxes between the SeAs. (A) Predicted red blood cell fluxes in the SeAs continue to decay with distance from the heart, when the model from [6] is used to parameterize phase skimming. (B) Hematocrit is predicted to increase with the distance from the heart but Hematocrit changes are not enough to compensate for the decrease in flow predicted by the hydraulic resistor network model. Bars show the hematocrits in the different SeAs, while the black reference line shows the mean hematocrit in the DA.

S4. MEAN FIELD MODEL FOR A TWO-VESSEL NETWORK

To understand the role of variations in the occlusive effect between SeA-vessels, we develop a continuum model on a reduced network that included only the DA and the first and last of the SeAs. The results of this analysis are summarized in the main text. Here, we describe in more detail the equations that are set up within the model, as well as their solution. The reduced network consists of 4 blood vessels indexed 1 (the segment of DA between the two Se), 3 (the segment of DA after the last Se, which connects directly to the PCV) and $2, 4$ being the two Se-vessels (Fig. 5A in main text). We define variables n_i, l_i, S_i, V_i, Q_i, R_{i0}, R_i $i = 1, 2, 3, 4$ to be the number of cells contained in vessel i, its length, cross-section area, volume, total flow rate, resistance when no red blood cells are present within the vessel, and resistance modified by the presence of cells following Equation (1) in the main text. Since our analyses from Section S3 suggest that phase separation effects are slight in these trunk microvasculature, we neglect them altogether, assuming constant hematocrit in each vessel. In the mean field formulation, the effect of this is to take $\frac{n_i}{V_i} = \rho, i = 1, 2, 3, 4$, where ρ is the constant concentration (# per volume) of red blood cells. ρ is related to the hematocrit (or volume fraction of red blood cells) by $\text{Hct} = \rho V_c$ where V_c is the volume of a cell. Thus the flux of red blood cells in vessel i is equal to ρQ_i. Finally we define $p_j, j = 1, 2$ to be the unknown pressures at the two branching points within the network (as in the model without feedbacks, the symmetry of the network allows us to assign the same pressure value, $p = 0$ at the points where the SeAs meet the DLAV and where the DA terminates at the tail of the fish). We make a continuum or mean field approximation for the effect of the red blood cells contained in each vessel. Specifically, we assume that red blood cells in each vessel are uniformly dispersed through that vessel. Then, in steady state we can balance the flux of cells into each vessel with the flux of cells out of each vessel. For example, for vessel 1, the flux of cells (number/time) out of the vessel is equal to the volume of blood leaving the vessel in unit time Q_1 multiplied by the density (number/volume) of cells: n_1/V_1. Similarly the flux of cells into the vessel is given by the total flux of cells entering the network through node 1, in unit time multiplied by the ratio in which flow is divided between vessels 1 and 2 ($\frac{Q_1}{Q_1+Q_2}$). Since the total number of red blood cells in the network is constant in the continuum approximation, the number of red blood cells entering the network in unit time must be equal to the number leaving,

i.e. $\frac{n_2 Q_2}{V_2} + \frac{n_3 Q_3}{V_3} + \frac{n_4 Q_4}{V_4}$. Thus in steady state:

$$\left(\frac{n_2 Q_2}{V_2} + \frac{n_3 Q_3}{V_3} + \frac{n_4 Q_4}{V_4}\right) \frac{Q_1}{Q_1 + Q_2} = \frac{n_1 Q_1}{V_1}. \tag{S3}$$

Similar conservation statements for each of the other 3 vessels in the network give:

$$\left(\frac{n_2 Q_2}{V_2} + \frac{n_3 Q_3}{V_3} + \frac{n_4 Q_4}{V_4}\right) \frac{Q_2}{Q_1 + Q_2} = \frac{n_2 Q_2}{V_2} \tag{S4}$$

$$\frac{n_1 Q_1}{V_1} \frac{Q_3}{Q_3 + Q_4} = \frac{n_3 Q_3}{V_3} \tag{S5}$$

$$\frac{n_1 Q_1}{V_1} \frac{Q_4}{Q_3 + Q_4} = \frac{n_4 Q_4}{V_4} \tag{S6}$$

Flux conservation at each node, plus the resistance-to-cell number relationship in Eqn. (1) from the main text then allows us to compute the fluxes Q_i. Specifically, suppose the fluid inflow is F into the first node. Then since flow rate is proportional to the pressure difference across a vessel:

$$Q_1 = \frac{p_1 - p_2}{R_1}, Q_2 = \frac{p_1}{R_2}, Q_3 = \frac{p_2}{R_3}, Q_4 = \frac{p_2}{R_4}. \tag{S7}$$

while conserving fluxes at the two nodes gives:

$$F = \frac{p_1 - p_2}{R_1} + \frac{p_1}{R_2} \tag{S8}$$

$$\frac{p_1 - p_2}{R_1} = \frac{p_2}{R_3} + \frac{p_2}{R_4} \tag{S9}$$

We can solve for the nodal pressures p_1, p_2 by linear algebra. Define a matrix determinant:

$$\Delta = \begin{vmatrix} \frac{1}{R_1} + \frac{1}{R_2} & -\frac{1}{R_1} \\ \frac{1}{R_1} & -(\frac{1}{R_1} + \frac{1}{R_3} + \frac{1}{R_4}) \end{vmatrix} = -\left(\frac{1}{R_1} + \frac{1}{R_2}\right)\left(\frac{1}{R_1} + \frac{1}{R_3} + \frac{1}{R_4}\right) + \frac{1}{R_1^2}. \tag{S10}$$

Then Cramer's rule gives:

$$p_1 = \frac{1}{\Delta} \begin{vmatrix} F & -\frac{1}{R_1} \\ 0 & -(\frac{1}{R_1} + \frac{1}{R_3} + \frac{1}{R_4}) \end{vmatrix} = \frac{-F(\frac{1}{R_1} + \frac{1}{R_3} + \frac{1}{R_4})}{\Delta} \tag{S11}$$

$$p_2 = \frac{1}{\Delta} \begin{vmatrix} \frac{1}{R_1} + \frac{1}{R_2} & F \\ \frac{1}{R_1} & 0 \end{vmatrix} = \frac{-\frac{F}{R_1}}{\Delta}. \tag{S12}$$

From the formulas for the nodal pressures p_i we can use Eqn. (S7) to calculate the fluxes in each vessel, Q_i:

$$Q_1 = \frac{-F}{\Delta R_1}\left(\frac{1}{R_3} + \frac{1}{R_4}\right),\ Q_2 = -\frac{F}{\Delta R_2}(\frac{1}{R_1} + \frac{1}{R_3} + \frac{1}{R_4}),\ Q_3 = \frac{-F}{\Delta R_3 R_1},\ Q_4 = \frac{-F}{\Delta R_4 R_1} \tag{S13}$$

Although these represent volume fluxes (volume/time), the cell flux in each vessel can then be computed by multiplying by the cell concentration, ρ. Using Equation (1) from the main text, we may rewrite $R_2 = R_{20} + \alpha_2 \rho V_2$, and so on.

To analyze flows within the network we focused on two measures of efficiency, (i) One is the ratio of the cells fluxes in the two SeAs:

$$\begin{aligned} \text{flux ratio} &= \frac{\rho Q_2}{\rho Q_4} = \frac{Q_2}{Q_4} = \frac{R_1 R_4}{R_2}\left(\frac{1}{R_1} + \frac{1}{R_3} + \frac{1}{R_4}\right) \\ &= \frac{\bar{R}_4 + V_4 \rho \alpha_4}{\bar{R}_2 + V_2 \rho \alpha_2}\left(1 + \frac{R_1}{R_3} + \frac{R_1}{R_4 + V_4 \rho \alpha_4}\right) . \end{aligned} \tag{S14}$$

(ii) We also compute the viscous dissipation within the network when the flux through all SeAs, i.e. $\rho(Q_2 + Q_4)$, is fixed. The dissipation for a single vessel is given by $D = Q^2 R$ where Q is the flow rate of the vessel, R is the resistance of the vessel. Thus the dissipation of the entire network is

$$D_{\text{network}} = \sum_{i=1}^{4} Q_i^2 R_i. \tag{S15}$$

For the aorta segments, we assume constant resistance (not strongly affected by the number of red blood cells) so

$$D_{\text{aorta}} = \frac{8\mu_{wb}}{\pi r_a^4} \sum_{i=1}^{2} l_{2i-1} Q_{2i-1}^2. \tag{S16}$$

In contrast the dissipation within SeAs depends on the number of cells traveling through them:

$$D_{\text{Se}} = \sum_{i=1}^{2} Q_{2i}^2 R_{2i} = \sum_{i=1}^{2} Q_{2i}^2 R_{2i0} + \sum_{i=1}^{2} Q_{2i}^2 n_{2i} \alpha_{2i} \tag{S17}$$

Substituting $n_i = \rho V_i$, we obtain:

$$D_{\text{Se}} = \frac{8\mu_{pl}}{\pi r_c^4} \sum_{i=1}^{2} l_{2i} Q_{2i}^2 + \rho \sum_{i=1}^{2} V_{2i} \alpha_{2i} Q_{2i}^2 \tag{S18}$$

and

$$D_{\text{network}} = D_{\text{aorta}} + D_{\text{Se}} = \frac{8\mu_{wb}}{\pi r_a^4} \sum_{i=1}^{2} l_{2i-1} Q_{2i-1}^2 + \frac{8\mu_{pl}}{\pi r_c^4} \sum_{i=1}^{2} l_{2i} Q_{2i}^2 + \rho \sum_{i=1}^{2} V_{2i} \alpha_{2i} Q_{2i}^2 \tag{S19}$$

which is Equation (6) in the main text.

S5. ESTIMATION OF OCCLUSIVE EFFECTS IN A 4 DPF ZEBRAFISH

To analyze the effect of occlusive feedbacks upon the distribution of red blood cell fluxes within the trunk vessels, we directly measured the occlusive feedback parameter α_c from Equation (1). Namely, for each vessel, we fit an equation for the resistance of the vessel as a function of the number, n of cells that it contains:

$$R = R_0 + n\alpha_c \tag{S20}$$

where R is the resistance of Se, R_0 is the resistance of the SeA when it contains no cells, which is given by Hagen-Poiseuille law calculated with plasma viscosity $\mu_{pl} \approx 1cP$, and α_c is the occlusive effect per cell. We directly measure the coefficient α_c by tracking red blood cells in a real zebrafish embryo. To do this me must convert Eqn. (S20) into an equation for velocity in the vessel. Here we expand our discussion of how this model was fit to the real data, and show the fits for each of the SeAs.

Since the resistance R is the ratio between the pressure drop Δp over the Se vessel and the flow rate Q in the vessel we have

$$QR = \Delta p. \tag{S21}$$

Plugging Eqn. (S20) into Eqn. (S21) we obtain:

$$R_0 + n\alpha_c = \frac{\Delta p}{Q} = \frac{\Delta p}{u\pi r^2}, \tag{S22}$$

where u is the mean plasma velocity and r is the radius of the vessel. Hence in the vessel $\frac{1}{u}$ is linearly related to n:

$$\frac{1}{u} = \frac{\pi r^2}{\Delta p}(R_0 + n\alpha_c), \tag{S23}$$

assuming that Δp is constant. Since in the theory developed in [7], red blood cells travel at the mean velocity of the plasma, we calculated $\frac{1}{u}$ by tracking by hand the cells traveling through the Se arterial network. If we regress $\frac{1}{u}$ against n then the slope a and the intercept b of the line satisfies

$$a = \frac{\pi r^2 \alpha_c}{\Delta p}, \quad b = \frac{\bar{R}\pi r^2}{\Delta p}, \tag{S24}$$

which allows us to calculate the resistance per cell α_c:

$$\frac{a\bar{R}}{b} = \alpha_c. \tag{S25}$$

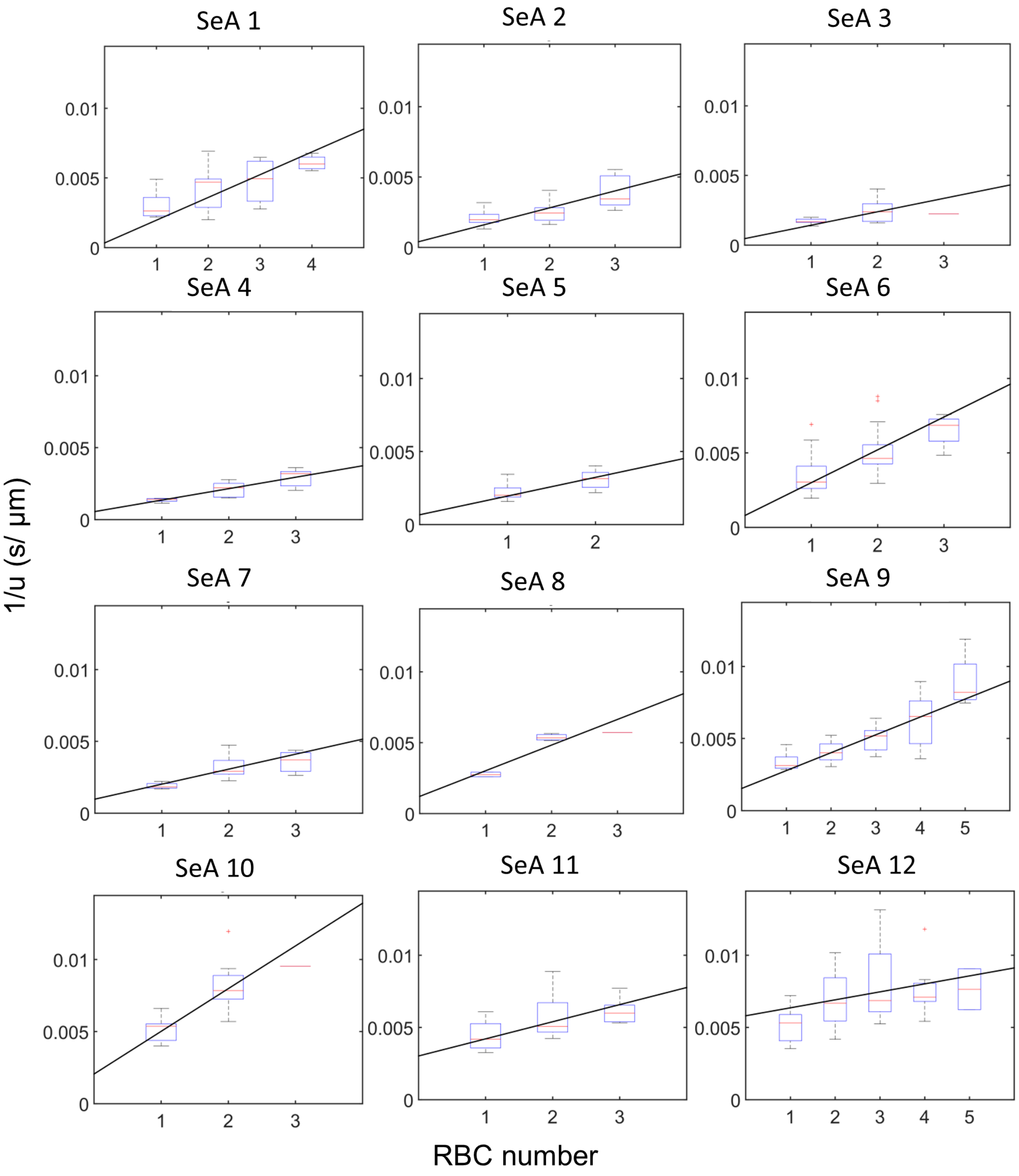

FIG. S2: Occlusive effects are measured in all 12 Se arteries in a 4 dpf zebrafish; we regress the reciprocal of the average velocity $\frac{1}{u}$ against the cell number n. Line: linear regression with intercept determined by the numerical solution with no cells.

Fig. S2 shows the experimental measurements and the regression. The maximum number of cells in a vessel is quite low due to the occlusive effect, which greatly decreases flow when

a vessel contains multiple cells. Therefore we decided to use the theoretical prediction of plasma velocity with no cells from Figure 1C in the main text to fit $1/v$ when $n = 0$, and thereby the pressure drop Δp. In applying the cell-free model we ascribed all Se vessels the same radius and also fixed the radii of all aorta segments. This reduces the noise caused by variation of radius but preserves the key feature of exponential decay. The resulting Fahraeus-Lindqvist effect coefficients α_c are shown in Fig. 2B in the paper. Note that there is considerable scatter in the mean velocity data shown in Fig. S2 (a single panel of this figure is displayed in the main text as Fig. 2A). This scatter is probably dominated by the complex stick-slip dynamics of red blood cells even when propelled by steady pressure gradients[7], and by the variation in the pressure drop Δp across each Se vessel over each cardiac cycle. Previous measurements have shown that flow rates in the aorta vary by a factor of 6 over a single heart beat[8]. By lumping together velocimetric measurements from different phases of the cardiac cycle, our data include unavoidable velocity variation, distinct from measurement error. However, our theory and fits extract the average values of Δp over a full cardiac cycle.

S6. EFFECT OF NETWORK PERTURBATION UPON RED BLOOD CELL PARTITIONING

Real zebrafish vascular networks, and microvascular networks generally vary from individual to individual[5, 9]. Some forms of anatomical variation lead to embryo death, while others do not affect embryo viability at all. We used the model of feedbacks due to vessel occlusion to determine whether uniform red blood cell fluxes could be achieved in two previously studied forms of vascular network variability.

A. Variation in spacing between intersegmental vessels

In real vascular networks the SeAs are not evenly spaced. In our model (and supported by visualization of the dsRed-tagged cell movements), we assume that the Se vessels alternate artery-vein-artery-. . . . Real trunk vasculature does not always follow this pattern of strict alternation; in fact arteries and veins can be ordered in many different ways, and the particular ordering of vessels seems to have little impact on embryo growth[5], we therefore

infer that it does not affect oxygen perfusion through the trunk. To investigate whether the feedback mechanism robustly uniformizes cell fluxes, independently of the ordering of arteries and veins, we simulated cell partitioning between SeA in zebrafish with large variations in SeA spacing. Specifically, we define a vector $\{Pa(i) : i = 1, ...11\}$ of normalized intersegmental distances. The entries of Pa are normalized such that $\sum_{i=1}^{11} Pa(i) = 11$. The lengths $l_{2i-1}, i = 1, ..., 11$ of the DA segments are then given by

$$l_{2i-1} = l_{\text{aorta}} Pa(i), \tag{S26}$$

where $l_{\text{aorta}} = 169$ μm is the mean Se spacing in a 4 dpf zebrafish. Fix the length of the last DA segment (between the final Se and the direct connection to the PCV) to be $l_{23} = 169$ μm. We create a network with high variation in the Se spacing by setting $Pa(2i-1) = 1.69, i = 1, \ldots, 6$ and $Pa(2i) = 0.169, i = 1, \ldots, 5$, so that successive spacings differ by a factor of 10. Just as in Fig. 4, we assume a linearly decreasing feedback strength (i.e. a linear form for the resistance per cell α_i), in which the resistance per cell in the i-th SeA is given by a formula:

$$\alpha_i = \frac{(\alpha_{\min} - \alpha_{\max})i}{n-1} + \alpha_{\max} - \frac{\alpha_{\min} - \alpha_{\max}}{n-1}, \quad i = 1, \ldots, n \tag{S27}$$

where $\alpha_{max} = 2.334 \times 10^{-5}$ g/μm^{4}s is the feedback strength within the first Se from the data and $\alpha_{min} = 1.01 \times 10^{-6}$ g/μm^{4}s is that of the last Se. α_{max} and α_{min} are obtained from linear regression on the measured feedback strengths (see main-text, Fig. 2C). We then used a direct numerical simulation of the cell dynamics in this network (see Materials and Methods in the main text) to calculate the partitioning of cells in the modified network. We estimate the uniformity of flows for each set of network parameters by computing the Coefficient of Variation (CV) of the cell flux. The CV is 0.2538 in the uneven spacing case, which indicates a lower uniformity compared to a network with the empirically determined Se spacings (with CV value 0.1815, see Fig. S3). However, if the feedback strength varies with distance along the DA, (i.e. with vessel distance from the heart, rather than simply being a function of vessel index), namely:

$$\alpha_i = (\alpha_{max} - \alpha_{min}) \frac{\sum_{j=i}^{n-1} Pa(j)}{\sum_{j=1}^{n-1} Pa(j)} + \alpha_{min} \tag{S28}$$

then the CV of cell fluxes under the same simulation conditions as were used to create Fig. 2, is 0.1827, which is almost identical to the unperturbed network.

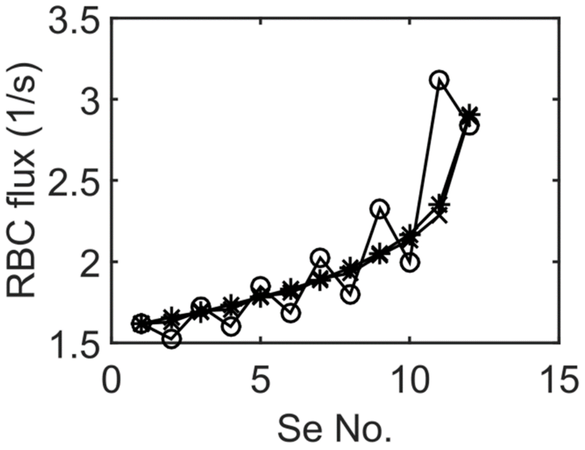

FIG. S3: Predicted cell fluxes in wildtype zebrafish due to variability in Se spacing variant. The wildtype cell fluxes (star) becomes oscillatory under variant spacing (circle), but shows similar overall uniformity. If the feedback variation is adjusted then uniform partitioning of cell fluxes is restored (cross).

B. DA-PCV shunt

Genetically modified mib^{ta52b} mutant zebrafish have altered differentiation of vessels into arteries or veins. In particular the mutant trunk vasculature includes a circulatory loop (shunt) between DA and PCV in the middle of the trunk [10]. mib^{ta52b} mutants die before two weeks post fertilization [10]. To simulate the effect of mib^{ta52b} upon the partitioning of cells through the zebrafish trunk vasculature we created a model of the network, by starting with the same wild type network geometry as in Fig. 1B in the main text but with the 6th SeA being assigned a radius 7 μm (identical to the aorta) and a length 17 μm, based on vessel measurements from [10]. The length of the DA vessel segments on either side of the shunt connection were set to be one half of the mean DA vessel segment length, mimicking the DA malformation seen around the shunt in real zebrafish [10]. The cell flux in the shunt, 39 s^{-1}, is much greater than the mean cell flux in all the SeAs, 0.3 s^{-1}. Because cells have smaller radii than the shunt connection, cells do not occlude this vessel, there is no negative feedback and cells are not redistributed to SeAs beyond the shunk (Fig. S4). We expect the low cell fluxes in the other vessels to be associated with low oxygen transport to the rest of the trunk, which may contribute to the lethality of the mib^{ta52b} mutation.

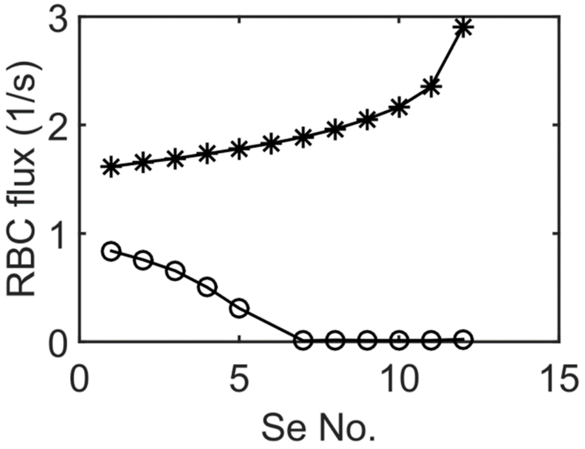

FIG. S4: Predicted cell fluxes in mib^{ta52b} mutant zebrafish. In this mutant, the DA and PCV are directly connected by a shunt, which creates a short-circuit in the network. A shunt introduced at the location of the 6th Se leads to lower and less uniform fluxes (circle) compared to wild type embryos (star), and there is almost no cell flux posterior to the shunt location.

[1] P. Rombough and H. Drader, J. Exp. Biol. **212**, 778 (2009).

[2] B. M. Weinstein, D. L. Stemple, W. Driever, and M. C. Fishman, Nat. Med. **1**, 1143 (1995).

[3] S. Kranenbarg, J. G. van den Boogaart, and J. L. van Leeuwen, Anim. Biol. **53**, 339 (2003).

[4] D. Salgado, C. Marcelle, P. D. Currie, and R. J. Bryson-Richardson, Dev. Biol. **372**, 1 (2012).

[5] S. Isogai, M. Horiguchi, and B. M. Weinstein, Dev. Biol. **230**, 278 (2001).

[6] A. R. Pries and T. W. Secomb, American Journal of Physiology-Heart and Circulatory Physiology **289**, H2657 (2005).

[7] T. Secomb, R. Hsu, and A. Pries, American Journal of Physiology-Heart and Circulatory Physiology **274**, H1016 (1998).

[8] M. H. Malone, N. Sciaky, L. Stalheim, K. M. Hahn, E. Linney, and G. L. Johnson, BMC biotechnology **7**, 40 (2007).

[9] B. W. Zweifach and H. H. Lipowsky, Circ. Res. **41**, 380 (1977).

[10] N. D. Lawson, N. Scheer, V. N. Pham, C.-H. Kim, A. B. Chitnis, J. A. Campos-Ortega, and B. M. Weinstein, Development **128**, 3675 (2001).
